# The Monitoring of *Mycoplasma gallisepticum* Minimum Inhibitory Concentrations during the Last Decade (2010–2020) Seems to Reveal a Comeback of Susceptibility to Macrolides, Tiamulin, and Lincomycin

**DOI:** 10.3390/antibiotics11081021

**Published:** 2022-07-29

**Authors:** Marco Bottinelli, Michele Gastaldelli, Micaela Picchi, Arianna Dall’Ora, Lorena Cristovao Borges, Ana Sofía Ramírez, Andrea Matucci, Salvatore Catania

**Affiliations:** 1 Mycoplasma Unit–SCT1-Verona, WOAH Reference Laboratory for Avian Mycoplasmosis, Istituto Zooprofilattico Sperimentale Delle Venezie, 37060 Buttapietra (VR), Italy; mgastaldelli@izsvenezie.it (M.G.); mpicchi@izsvenezie.it (M.P.); adallora@izsvenezie.it (A.D.); lcristovao@izsvenezie.it (L.C.B.); amatucci@izsvenezie.it (A.M.); scatania@izsvenezie.it (S.C.); 2 Unidad de Epidemiología y Medicina Preventiva, Instituto Universitario de Sanidad Animal y Seguridad Alimentaria, Universidad de Las Palmas de Gran Canaria, 35413 Arucas, Spain; anasofia.ramirez@ulpgc.es

**Keywords:** *Mycoplasma gallisepticum*, MIC, antimicrobial susceptibility, macrolides, poultry

## Abstract

*Mycoplasma gallisepticum* (*Mg*) is a highly contagious avian pathogen responsible for significant economic losses for the poultry industry. In some circumstances, antimicrobial treatment is useful to contain clinical signs of *Mg* infection in birds. However, antimicrobial resistance emergence is now common among animal pathogens, becoming a worldwide health concern. The collection of minimum inhibitory concentration (MIC) data is fundamental for an appropriate antimicrobial use and for fighting antimicrobial resistance emergence. However, MIC data can only be generated in specialized laboratories, and therefore they are not regularly available. MICs of 67 non-vaccine-derived *Mg* isolates collected in Italy between 2010 and 2020 were obtained. Although 79.1% of the *Mg* isolates showed enrofloxacin MICs ≥ 8 µg/mL, a statistically significant trend toward low MICs of erythromycin, tylosin, tilmicosin, spiramycin, tiamulin, and lincomycin was observed, indicating a comeback to susceptibility of *Mg* toward these drugs. Doxycycline proved to be slightly more effective than oxytetracycline. The present study shows that *Mg* changed its susceptibility toward many of the drugs most commonly used for its containment over a ten-year period.

## 1. Introduction

Nowadays, chicken is the animal species most commonly farmed in the world (three for every human), and poultry is one of the most important food industries [1,2]. In fact, chicken meat represented 35% of the world’s meat production in 2020 [3]. *Mycoplasma gallisepticum* (*Mg*) is a well-known avian pathogen able to cause a decidedly contagious, chronic respiratory disease in industrial poultry animals. *Mg* belongs to the class *Mollicutes*, which is composed of fastidious, minimalist, wall-less microorganisms. Despite being simple in appearance, *Mg* is responsible for significant economic losses in the poultry industry [4], causing increased condemnations at processing, downgrading of carcasses, reduction of egg production, feed conversion ratio, and egg hatchability [4]. 

Even though the epidemiology of avian mycoplasma infections continues to be puzzling for both scientists and poultry industry experts [5], it is known that these organisms are transmitted both horizontally, among susceptible hosts, and vertically, *in ovo* [4]. Therefore, the control of mycoplasma infections can be achieved through prevention, vaccination, or medication of the affected animals. The prevention of the infection is made through implementation of biosecurity measures with the ultimate aim of maintaining the flocks—especially breeder stocks—free from mycoplasmas [6]. In situations where this goal is not attainable, vaccination of the animals is a viable option and, currently, many vaccines are available for use in the different poultry production categories [4,6]. Although *Mg* prevalence has been significantly decreasing in the Western countries over the last 20 years [7], consistently applied monitoring systems regularly detect outbreaks of disease, especially in densely populated poultry areas (DPPAs) [5]. 

As soon as an outbreak occurs, proper medication of the animals is usually effective in reducing clinical signs and in curbing the spread of the microorganism among the different barns within a single farm [8]. However, because mycoplasmas are slow-growing organisms, the choice of the antimicrobial molecules for the treatment is often empirical rather than being addressed on the basis of in vitro susceptibility data obtained in specialized laboratories [9]. The range of antimicrobials available for treating *Mg* infections is narrowed by the intrinsic resistance of *Mollicutes*, which is linked to the absence of antimicrobial targets in the bacterium. For instance, *Mollicutes* are insensitive to betalactams, glycopeptide antibiotics, and bacitracin because they lack cell walls. They are not sensitive to sulphonamides and trimethoprim because they do not possess any enzyme for folic acid synthesis. In addition, due to conservative mutations in RNA polymerase *Mollicutes* are resistant to rifampicin [10]. Most antimicrobials have no more than a bacteriostatic effect against mycoplasmas, generally resulting in an unsatisfactory, slow elimination of these organisms from the infected tissues. An effective treatment could be achieved using antimicrobial agents that penetrate cells (fluoroquinolones, florfenicol, lincosamides, macrolides, or tetracyclines) and through prolonged administration of the drug over time [9]. Considering this, the risk of selection of resistant strains is concrete, and remedial actions are certainly needed. The evidence to support this claim can be found in the literature, wherein several reports of *Mg*-resistant strains are available, as well as in studies on the in vitro-resistance development of avian mycoplasma species [11]. It is noteworthy that *Mollicutes* of animal origin possess a greater number of mutations in genes coding for target proteins compared with *Mollicutes* of human origin [11]. This finding can be due to the extensive usage of antimicrobials in animal production [12], which is also accompanied by the risk of antimicrobial resistance (AMR) development in commensal organisms, a worldwide health concern [13]. Actions against AMR have been taken by the Italian government through the endorsement of national plans, which aimed at reducing the antimicrobial consumption in poultry production. The overall reduction of the antimicrobial use in broilers and turkey has been documented already [14]; however, recent data on *Mg* MICs is limited even though it is fundamental for monitoring AMR. 

This study had multiple aims. The first one was to collect minimum inhibitory concentration (MIC) values of non-vaccine-derived *Mg* strains isolated in Italy between 2010 and 2020. Secondly, by utilizing appropriate statistical approaches, the authors aimed at (1) detecting any significant antimicrobial susceptibility variation over time toward the most common antimicrobials used for *Mg* infection treatment, and (2) creating susceptibility trend models. The importance of the research output derives from the impossibility to have MIC data readily available for veterinarians when approaching a recently diagnosed mycoplasma outbreak, which usually results in empirical antimicrobial administration to the animals. 

## 2. Results

On a total of 143 *Mg* isolates, 67 isolates were eligible for enrollment in the study. The MIC values obtained during the study and the antimicrobial dilution ranges are summarized in Table 1. The graphical representation of the isolate distribution along the dilution range of each drug is reported in Figure S1.

MIC values of the *Mg* type strain MG ATCC 15302 showed consistency throughout the study, indicating a high reproducibility of the tests performed. The MIC values of each *Mg* isolate revealed no discrepancy among replicates of the test.

Except for doxycycline, tiamulin, and florfenicol, MIC values of all drugs showed a bimodal distribution. Six *Mg* isolates showed high oxytetracycline MIC values (≥32 µg/mL). A three-dilution difference was observed between the MIC distributions of the two tetracycline drugs, with doxycycline being more effective than oxytetracycline. Fifty-three out of sixty-seven (79.1%) Italian *Mg* isolates showed enrofloxacin MIC values ≥ 8 µg/mL. Both MIC50 and MIC90 values for enrofloxacin were higher than the highest dilution present on the plate (>16 µg/mL). The Italian *Mg* sample showed different behaviors towards the macrolide drugs tested. Against erythromycin, the *Mg* isolates showed either very low (≤0.5 µg/mL; 39/67–58.2%) or very high (>8 µg/mL; 28/67–41.8%) MIC values. The same pattern was observed for tilmicosin and spiramycin too, even though some more variability of MICs was observed for these two drugs. In contrast, a higher variability in tylosin MICs was recorded (see Supplementary material Figure S1). The *Mg* isolates characterized by higher macrolide MICs were collected more frequently during the first half of the study timeframe. Seven *Mg* isolates collected between 2010 and 2016 showed high tylosin MICs (≥8 µg/mL), high tilmicosin MICs (>32 µg/mL), and high lincomycin MICs (≥16 µg/mL) at the same time. All *Mg* isolates showed tiamulin MICs ≤ 0.25 µg/mL, with MIC50 and MIC90 values being 0.015 µg/mL and 0.125 µg/mL respectively. Half of the Italian *Mg* sample gathered on the right of the lincomycin graph, being characterized by high MICs (≥16 µg/mL). Florfenicol MICs ranged between ≤0.5 and 4 µg/mL.

**Figure 1:**
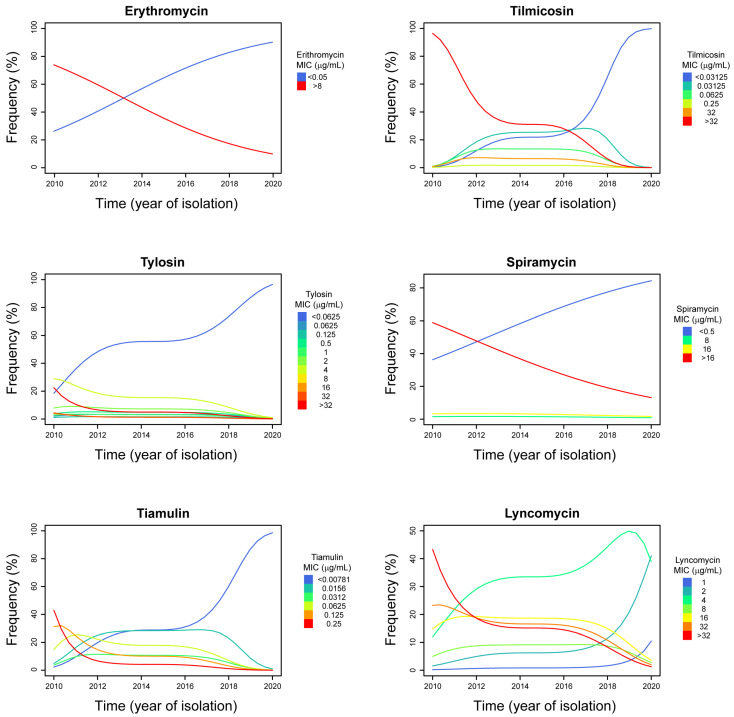
MIC classes frequency trends during the study time (2010–2020). The year of isolation of the *Mg* isolates is reported on the horizontal axis of abscissas. The percentage of isolates that were inhibited by each of the MICs is reported on the vertical axis of the ordinates. A different colour was assigned to each MIC concentration (intended as class), as indicated on the left of each data graphic. The lines in the data graphic boxes indicate the trend of each MIC class over time.

The results of the linear-by-linear association test made on all drugs (except doxycycline) are reported in Table 2. A statistically significant time-dependent variation of MIC frequencies was observed for erythromycin, tilmicosin, tylosin, spiramycin, tiamulin, and lincomycin. The presence of an either positive or negative correlation between the ordinal variables is indicated by the sign (+/−) of the *Z*-value. In order to investigate the shape of the relation between the frequency of the different MIC classes and the year of isolation, logistical and proportional odds models were constructed respectively for erythromycin and for those antimicrobials whose linear-by-linear test *p*-value was <0.05. The models are presented in Figure 1, whereas the parameter estimates with relative standard errors, Wald statistics, and *p* values of the models are reported in Supplementary Materials Tables S1–S6. 

Taking into account the size of our dataset and the strength of the observed relationships, the predictive value of erythromycin, lincomycin, spiramycin, and tylosin models is lower compared to that of tilmicosin and tiamulin models. Therefore, the MIC class trends evidenced in those models have a lower predictive power, although they are statistically significant. 

Except for spiramycin, it is possible to observe a clear biphasic pattern of the MIC class trends in the proportional odds models generated (see Figure 1). High MIC classes frequency decreased markedly from 2010 to 2012–2014, whereas a further marked reduction was observed again starting from 2016–2018.

## 3. Discussion

*Mg* is the most pathogenic of the avian mycoplasmas, responsible for significant losses for the poultry industry [4]. Even though containment tools, such as biosecurity measures and vaccination [6], are available, the high capacity of the pathogen to adapt to the industrial poultry system environment and to evade the immune system [15,16] allow it to persist successfully in DPPAs. In fact, complete control of *Mg* infection is hard to achieve due to the presence of large poultry populations, multiage farms, and several possible interconnections (personnel, feed truck, etc.) between the meat and the layer sectors [6]. Therefore, antimicrobial treatment can be as useful as beneficial when managing an outbreak in certain circumstances, bearing in mind that long-term use of antimicrobial drugs can lead to AMR emergence. The antimicrobials considered to be effective against mycoplasmas, and therefore the most widely used, are tetracyclines, macrolides, pleuromutilins, and fluoroquinolones [6,11,13,17,18]. The fact that only fluoroquinolones have a bactericidal effect whereas the others generally show only a bacteriostatic one could explain why mycoplasma infections are slowly responsive to treatment [9], further raising the risk of AMR emergence. Considering that it is not easy to isolate mycoplasmas and the long time-to-result for MIC assessment [19], most of the antimicrobial treatments applied to the animals are usually empirical rather than being prescribed based on actual susceptibility data [9]. Therefore, monitoring MICs in mycoplasmas remains crucial for the detection of AMR emergence caused by improper use of antimicrobial drugs.

It is generally accepted that AMR mechanisms in *Mollicutes* are mainly attributable to point mutations in genes whose products are targeted by the drugs [11,20]. However, in some cases it can occur that no mutation is found, suggesting the involvement of uncharacterized mechanisms [20]. Thus, the sole molecular screening for mutations in known target genes is not enough for AMR detection in mycoplasmas. The need for a standardized MIC determination method for mycoplasmas is thus real; it is fundamental to obtain reproducible data that allow comparing observations from different laboratories. The lack of interpretation criteria (cutoff values for sensitivity/resistance) for some mycoplasma-active antimicrobials makes it hard to determine the in vivo effectiveness of the drugs on the basis of in vitro susceptibility profiles. Therefore, the authors preferred to indicate the MIC values as low, intermediate or high, minimizing the use of the terms “sensitive”, “intermediate”, and “resistant”. As also recommended by Taiyari and collaborators [21], MIC tests were carried out by following the recommended procedure present in the literature [22]. In this way, our data can be compared with many of those previously published and therefore be potentially useful for scientists, veterinarians, and poultry industry specialists living in other parts of the world.

The class of tetracyclines is the one with the highest use in veterinary medicine, especially in food animals for which tetracyclines are considered as first-line drugs [23]. According to what is reported in the literature [11], most *Mg* isolates should be susceptible to tetracycline drugs. The Italian *Mg* sample distribution along the drug dilutions on the *x*-axis (see Table 1 and Figure S1) possibly indicates the absence of resistance selection towards these molecules except for six strains that showed high oxytetracycline MIC values (≥32 µg/mL). A recent review of the data presented in 23 studies investigating the MIC values of *Mg* obtained in 17 countries around the world [21] evidenced that 47% of the *Mg* strains tested were resistant to oxytetracycline. The tetracyclines are amphoteric compounds that are not readily accumulated in eukaryotic cells, in which *Mg* is able to enter and “hide” [23,24,25]. Indeed, it is reported that to control *Mg* infection in birds, a prolonged administration of oxytetracycline (250 ppm) in feed is required [26]. The mutations linked to tetracycline resistance are more commonly selected and maintained in microorganisms like *Mollicutes*, which do not possess a high number of ribosomal operons [11,27,28,29,30], and for this reason it can be hypothesized that the subinhibitory concentrations of oxytetracycline reached within the host cells could favor adaptive resistance in *Mg*. Differently, doxycycline, a second-generation tetracycline, possesses a higher liposolubility that lets it enter and accumulate in eukaryotic cells. Moreover, doxycycline is characterized by a time-dependent bactericidal effect against *Mg* [31]. We observed that the nine *Mg* isolates with high oxytetracycline MICs (≥16 µg/mL), which should be considered as resistant according to the breakpoint values suggested by Hannan and collaborators [22], showed lower doxycycline MICs. In general, we observed a three-dilution difference between oxytetracycline and doxycycline MICs. Therefore, it can be proposed to test only one of the two tetracycline molecules to check the behavior of a certain isolate against this antimicrobial class. Lastly, it is interesting to note that higher-than-usual doxycycline tissue concentrations have been observed in animals experimentally infected with *Mg* and exposed to enrofloxacin traces through drinking water [32]. This situation is not far at all from reality because both drugs have a broad spectrum of activity and are widely used in the poultry industry [26,33]. Therefore, it can be speculated that a possible enrofloxacin-enhanced effect of doxycycline against *Mg* in vivo might have hindered the development of resistance against this compound on the field. 

The fluoroquinolone antimicrobial class, to which enrofloxacin belongs, comprises compounds that are well-absorbed orally, penetrate almost any tissue and cell in the body, and exhibit a concentration-dependent bactericidal effect at appropriate concentrations [33]. This latter characteristic makes one suppose that fluoroquinolones would have the benefit of preventing AMR emergence, especially if therapies are based on MIC data. However, resistance to fluoroquinolones is frequently detected in bacteria isolated from animals [34]—mycoplasmas included [11,35]—to the point that certain compounds have been withdrawn from the market in some countries [33]. Resistance toward fluoroquinolones is generally the result of point mutations of target genes [20]. Actually, it is precisely the mechanism of action of these drugs that increases the overall DNA mutation frequency in bacteria [33,36,37], which, however, would theoretically create recessive mutations. On the contrary, as can also be observed in our data, although resistance to enrofloxacin is generally achieved at a lower speed compared to other drugs [38,39], resistant isolates persist in the poultry industry and do not reverse their susceptibility toward these compounds. In fact, most (79.1%) of the Italian *Mg* sample showed enrofloxacin MICs ≥ 8 µg/mL, and both MIC50 and MIC90 values were higher than the highest drug dilution tested on plate (>16 µg/mL). In the literature, it is reported that enrofloxacin is the molecule toward which resistance has been detected with highest frequency in *Mg* isolates that were collected between 1993 and 2018 in different countries of the world [21]. *Mg* isolates collected in different geographical locations before 1997 showed low MIC50 (0.05 µg/mL) and MIC90 (0.1 µg/mL) values for enrofloxacin [40]. In Israel, the *Mg* isolates collected between 1997 and 2003 showed enrofloxacin MIC values ≤0.5 µg/mL, whereas a marked decrease in susceptibility was recorded in 2005–2006 [41]. During another Israeli study, 79% of the strains isolated between 2006 and 2010 revealed to be resistant to enrofloxacin [42]. A survey conducted on *Mg* isolates collected between 2014 and 2016 indicated that *Mg* isolates from the UK had a lower MIC90 value (0.12 µg/mL) compared to those collected in Italy (8 µg/mL) and Spain (16 µg/mL) [43]. Unfortunately, recent European data on *Mg* MICs is limited for further comparison, but the increasing resistance to enrofloxacin is of great concern. Last but not least, most *Mg* isolates collected in Southeast Asia between 2018 and 2019 showed enrofloxacin MIC values ranging between 1.25 and 5 µg/mL and very high tilmicosin MIC values (between 16 and >64 µg/mL) [44], indicating that susceptibility certainly varies by geographical region and consequently by poultry-management system. In fact, antimicrobial usage can vary considerably both within and among continents [45].

The macrolide class of antimicrobials comprises molecules with a central 12- to 16-membered lactone ring that are able to inhibit bacterial protein synthesis [46]. The macrolides most commonly used in poultry include erythromycin, tylosin, and tilmicosin, which are available for administration either in the feed or in the drinking water [17]. Unlike *M. synoviae* (*Ms*) [39,47], *Mg* is not intrinsically resistant to erythromycin and, generally, most *Mg* isolates are susceptible to macrolides [11]. In our study, we observed two distinct *Mg* groups showing either very low (≤0.5 µg/mL) or very high (>8 µg/mL) erythromycin MIC values. Curiously, the logistic model revealed that there is a significant trend toward low erythromycin MIC classes from 2010 to 2020, meaning that it is very likely that current *Mg* strains are susceptible to this compound. Also, the *Mg* isolates with low erythromycin MIC values were very susceptible to the other macrolides tested (spiramycin, tylosin, and tilmicosin) as well.

Tylosin, first developed at the end of the 1950s, is not as active as erythromycin against the majority of bacteria [46], but it has been historically indicated as one of the most effective antimicrobials for treating mycoplasma infections in poultry [6,18]. Moreover, tiamulin, a semisynthetic derivate of tylosin developed almost 40 years later, has proven effective against mycoplasmas—including *Mg* [48]—and it has also been used to treat other bacterial infections caused by *Pasteurella multocida* and *Ornithobacterium rhinotracheale* [49,50,51,52,53]. In the literature, there are reports of high MIC values of erythromycin, tylosin, and tilmicosin in *Mg* isolates collected in different countries before 2011 [40,42,54,55,56,57,58,59]. However, it must be mentioned that these values are generally lower compared to those obtained during our study. Gerchman and collaborators [42] reported that 50% of *Mg* Israeli isolates collected between 1997 and 2010 actually showed MIC50 values of and tilmicosin ≥ 10 µg/mL. However, since Gerchman and collaborators did not expose their *Mg* isolates to tilmicosin concentrations higher than 10 µg/mL, we cannot know if their actual MIC was similar to that of our *Mg* isolates, which is ≥32 µg/mL. Engagingly, the tylosin MICs obtained during our study showed a clear bimodal distribution; in fact, we found 28 isolates with high to very high tylosin MIC values (between 0.5 and >32 µg/mL). This group comprises isolates collected between 2010 and 2014 in most cases. Only four of these isolates were collected in 2016, and two were obtained in 2018, indicating a greater presence of tylosin resistance during the first part of the decade. The proportional odds analysis eventually confirmed that there is a statistically significant trend toward low tylosin MIC classes; a true comeback to susceptibility toward this compound has been occurring among Italian *Mg* isolates. The same phenomenon has been observed for both tilmicosin and spiramycin MIC classes too. The authors do not know why the Italian *Mg* isolates changed their antimicrobial susceptibility during the last decade, but they have come up with some hypotheses. First of all, it is logical to think that what we observed can be the result of a reduction of the antimicrobial selective pressure on field. Actually, starting from 2010, *Mg* MIC data have been more and more available among veterinarians and poultry sector specialists in Italy. This is because there has been an increased demand from this business sector for *Mycoplasma* isolation and MIC testing, whereas specific oral communications at poultry-industry conferences were made at the same time. In this way, the usage of enrofloxacin and macrolides has been discouraged because MICs indicated a low responsiveness of *Mg* to the antimicrobial treatment. Until now, however, we observed a comeback to susceptibility towards macrolides only. It is known that resistance to macrolides is more rapidly developed than that to enrofloxacin in vitro [38,39]. This is due to the fact that resistance to macrolides is the result of point mutations on the 23S rRNA (domain V), whereas enrofloxacin-resistance development requires multiple mutation in up to four specific genes (*gyrA*, *gyrB*, *parE*, and *parC*) involved in the supercoiling of DNA [20]. Therefore, it can be speculated that the rate of resistance acquisition is comparable to that of its loss. Another hypothesis is that mutations conferring macrolide-resistance would come with drawbacks, such as a reduced fitness of the mutant isolate. This occurrence, which has already been observed for *Helicobacter pylori* [60,61] and proposed for *Ms* [47], makes the mutant strains advantaged in surviving only in the presence of macrolide-selective pressure. In fact, as soon as the antimicrobial is removed, the non-mutant isolates, having a higher fitness, can overgrow to the point that the mutant strains disappear. This would explain the comeback to susceptibility observed among the Italian *Mg* isolates, but further studies are surely needed to confirm or reject this intriguing hypothesis.

As with erythromycin, our data revealed a bimodal distribution of the isolates for spiramycin MICs. In fact, we observed a population (40.3%) of isolates showing high spiramycin MIC values (≥8 µg/mL), most of which had MIC values ≥ 16 µg/mL. Although being a 16-membered macrolide as tylosin and tilmicosin, spiramycin is not as effective against mycoplasmas. However, this compound gets highly concentrated in tissues, reaching 25–60 times serum concentrations. Therefore, spiramycin is paradoxically less active in vitro than in vivo [46]. This phenomenon could likely create biases when interpreting MIC results. The proportional odd analysis revealed a significant trend toward low MIC classes from 2010 to 2020 for spiramycin too, confirming the increased susceptibility to macrolides of the Italian *Mg* isolates over time. Interestingly, except for spiramycin, it is possible to observe a clear biphasic pattern of MIC-class trends in the proportional odds models generated in this study (see Figure 1). High MIC-classes frequency decreased markedly from 2010 to 2012–2014, whereas a further marked reduction has been observed again starting from 2016–2018. Low MIC-classes frequencies moved in the opposite direction. This occurrence could be linked to the research output generated by Matucci and collaborators [62], which highlighted the appearance of new *Mg* genotypes in Italy of unknown origin, probably caused by diverse events occurring in the poultry industry scenario, such as the H7N7 HPAI epidemic in late summer 2013 [63] or the eggshell apex abnormality outbreaks [64,65]. It is possible that the increased biosecurity measures applied on farm due to Avian Influenza virus circulation may have hindered mycoplasma spread among poultry farms. In the Italian industrial poultry system, vertical transmission of pathogenic mycoplasmas is negligible due to the maintenance of mycoplasma-free breeder stocks. Therefore, horizontal transmission of *Mg* is more likely to occur, as confirmed by the isolation of some genotypes in certain areas only [62]. A reduced circulation of the poultry-industry-related *Mg* strains within the poultry industry itself may have favored the diffusion of other *Mg* strains, such as those coming from backyard poultry. Overall, the presence of new *Mg* genotypes could have possibly contributed to the change of antimicrobial susceptibility observed in the Italian *Mg* population, although further studies are certainly needed to confirm this hypothesis. Correlations between MIC values and *Mg* isolates’ origin (industrial poultry sectors) have been investigated, but nothing significant was found. Therefore, biases caused by differences in antimicrobial usage among poultry sectors (e.g., layer sector) have been excluded. Lastly, it is likely that the national plans endorsed by the Italian Ministry of Health in 2015 and 2017 with the aim of reducing antimicrobial usage in poultry production and antimicrobial resistance have been contributing to the comeback to susceptibility of *Mg* isolates observed in this research work. Some effects of these national plans were described by Caucci and collaborators [14], who reported a decreasing trend of antimicrobial usage in broilers and turkeys during the years 2015–2017 in Italy.

Pleuromutilins and lincosamides are structurally distinct antimicrobial compounds that, however, share many properties. In fact, they are high liposoluble, they distribute widely in the body, and are able to pass through cellular barriers. Within prokaryotic cells they act in the same way as macrolides, interfering with bacterial protein synthesis [66]. Pleuromutilins, which tiamulin belongs to, possess outstanding activity against mycoplasmas, even better than that of macrolides. The administration of tiamulin in the drinking water has shown to be successful for the control of *Mg* infections [67], although these antimicrobials are more widely used in swine. According to what is reported in the literature [68,69,70], most of swine respiratory mycoplasmas have MIC values ranging between 0.064 and 0.5 µg/mL. Twenty-three (34.3%) of the Italian *Mg* isolates had MIC values falling within this range, whereas the majority showed to be sensitive to lower tiamulin concentrations. Our results are in agreement with those reported in the literature, namely that *Mg* MIC values for tiamulin are generally lower than MICs for other antimicrobials tested in vitro [11]. This finding could be explained by the fact that tiamulin is carefully used in poultry due to adverse effects in certain circumstances. In fact, tiamulin can interact with ionophores (e.g., monensin, lasalocide, salinomycin, etc.) causing growth depression, ataxia, paralysis, or even death. Therefore, ionophore drugs should not be administered to the animals during at least five days before/after the treatment with tiamulin [66]. On the basis of these considerations, we could assume that selective pressure made by tiamulin in the industrial poultry system is negligible. As with the macrolides, bacteria can become resistant to tiamulin following chromosomal mutation events. Even though the resistance emergence rate is much lower compared to that of tylosin, resistance towards pleuromutilins emerges quite quickly, at least in vitro. Interestingly, a one-way cross-resistance with tylosin exists: tylosin-resistant mycoplasmas show a slightly higher resistance to tiamulin, whereas tiamulin-resistant mycoplasmas are totally resistant to tylosin. Although objective interpretation of *Mg* MICs is not feasible due to the lack of resistance breakpoint values, we noticed that three out of four *Mg* isolates showing the highest tiamulin MIC value recorded during the experiment (0.25 µg/mL) were totally resistant to tylosin (MIC >32 µg/mL) (see Supplementary Materials). Lastly, it is interesting to note that a difference exists between the presented *Mg* MICs and those of *Ms* Italian isolates collected between 2012 and 2017. In fact, it seems that *Mg* is more susceptible to tiamulin (MIC50 = 0.015 µg/mL; MIC90 = 0.125 µg/mL) compared to *Ms* (MIC50 = 0.5 µg/mL; MIC90 = 1 µg/mL) [47]. The fact that MIC90 value for *Ms* is ten times higher than that for *Mg* is surprising, especially in light of the fact that these two species share their biological niche and they are supposedly exposed to the same antimicrobial selective pressure. In contrast, the Italian *Mg* isolates showed very high lincomycin MIC values (MIC50 = 16 µg/mL; MIC90 > 32 µg/mL), compared to those of the Italian *Ms* isolates (MIC50 ≤ 0.5 µg/mL; MIC90 = 2 µg/mL).

Lincomycin, the only lincosamides drug approved for the use in poultry, is a moderate spectrum antimicrobial that showed to be effective in treating mycoplasma infections in poultry [17,66,71,72]. It is usually sold in combination with spectinomycin, which results in a marginally enhanced effect against mycoplasmas, at least in vitro. Half of the Italian *Mg* sample gathered on the right of the lincomycin MIC graph (see Figure S1), showing high MICs (≥16 µg/mL). Resistance to lincosamides occurs more commonly as cross-resistance to macrolides, lincosamides, and streptogramin group B (MLSB resistance). This phenomenon is due to the fact that the macrolide binding sites on the 50S ribosomal subunit overlap with those of the lincosamides [46]. On a total of 34 *Mg* isolates with high lincomycin MIC values, 17 (50%) showed very high tilmicosin MIC values (≥32 µg/mL) and were not inhibited by the highest concentration of spiramycin (16 µg/mL) tested. Moreover, these *Mg* isolates showed MIC of tylosin ≥ 4 µg/mL, the value that indicates resistance towards this drug according to Hannan’s guidelines [22]. It has to be said that standard MIC tests allow the detection of only those isolates with constitutive MLSB resistance, characterized by the co-presence of high MICs for the different antimicrobials. In fact, the dissociated inducible kind of MLSB resistance is revealed only when the isolate is exposed to macrolides [66]. However, as with the macrolides, the proportional odds analyses revealed a statistically significant trend towards low MIC-classes for lincomycin too. Therefore, it can be assumed that we assisted to the disappearance of constitutive MLSB resistance in the Italian *Mg* population, even though genome analysis is needed to confirm it.

Florfenicol is a fluorinated analog of thiamphenicol approved for the use in food animals. It is a potent inhibitor of microbial synthesis binding irreversibly to a specific site of the 50S ribosomial subunit [73]. It is largely used in swine but not approved for use in poultry in Italy. Thiamphenicol is a phenicol drug approved for use in poultry that is less active than florfenicol, which instead may be more bactericidal [74]. The Italian *Mg* sample showed florfenicol MICs ranging between ≤0.5 and 4 µg/mL, a pattern that is identical to that of *M. hyorhinis* [75] but higher than that of *M. hyopneumoniae* [70]. The florfenicol MICs we obtained indicate a good efficacy of this drug against *M. hyorhinis* according to that proposed by Bekó and collaborators [75]. Again, it interesting to note that both Italian *Mg* and *Ms* isolates [47] show the same florfenicol MICs distribution, and that this data is in accordance with another study conducted by Gharaibeh and Al-Rashdan in 2011 [55]. 

A limitation of this research work is the lack of information on the history of antibiotic treatments given to the animals prior to *Mycoplasma* detection. Moreover, because custom-made commercial MIC plates were used, it was not possible to further investigate the extent of antimicrobial sensitivity of the isolates beyond the lowest and the highest drug concentrations present on the plates.

## 4. Materials and Methods

### 4.1. Mycoplasma gallisepticum Isolates

A total of 143 *Mg* isolates belonging to the Mycoplasma unit strain collection of the Istituto Zooprofilattico Sperimentale delle Venezie (IZSVe) collected in Italy between 2010 and 2020 were used for this research work. The isolates were collected from different poultry sectors (broiler, layer, turkey) and various avian species (chicken, turkey, guinea fowl, goose, pheasant, peacock). Only one isolate per single outbreak was included in the study group in order to avoid any duplicate. Each *Mg* strain was isolated from tracheal swabs collected from suspected or known infected animals. Information relative to the *Mg* isolates is reported in Table S7.

### 4.2. Mycoplasma gallisepticum Cultivation

Tracheal swabs collected from the animals were immersed and then shook in a selective culture broth (Avian Mycoplasma Liquid Medium, Mycoplasma Experience^®^, Reigate, UK). The culture broths were subsequently sent to the IZSVe Mycoplasma Unit for *Mycoplasma* spp. culturing. Cultivation performed following our internal procedure. Briefly, the culture broths were incubated at 37 ± 1°C under controlled atmosphere supplemented with 5% CO_2_ for up to 21 days. During the incubation period, the culture broths were visually inspected every day to detect any colour change (from orange to yellow) and/or any cloudiness. In case a modification of the culture broth appearance occurred or after 14 days without modifications, a 12-µL aliquot of the broth was then placed on a plate of avian mycoplasma agar (Mycoplasma Experience^®^, Reigate, UK), and the plate was incubated as described above. The plates were checked every day for the detection of any *Mycoplasma* colony. Samples without modifications seeded on agar were considered as negative in case no colony on agar was observed after 7 days of incubation. 

### 4.3. Mycoplasma gallisepticum Identification and Genotyping

For *Mycoplasma* species identification, the Maxwell^®^ 16 Blood DNA Purification Kit (Promega Italia Srl, Milano, Italy) was used for the extraction of the genetic material from a 300-µL aliquot of each positive suspect broth. The extracted DNA underwent a 16S-rDNA PCR and denaturing gradient gel electrophoresis (DGGE) as described in the literature [76]. With the purpose of genotyping the *Mg* isolates, *mgc2* sequence typing was carried out as reported by Matucci and collaborators [62]. Briefly, the *mgc2* gene was amplified as described in the literature [77,78]. The PCR products obtained were cleaned up by utilizing the Performa DTR Ultra 96-well kit (Edge BioSystems, Gaithersburg, MD, USA) and then sequenced by using BigDye Terminator v3.1 cycle sequencing kit (Applied Biosystems, Foster City, CA, USA) in a 16-capillary ABI PRISM 3130xl genetic analyzer (Applied Biosystems, Foster City, CA, USA). The Bioedit software 7.2.6.1 was used for assembling and editing of sequence data. Each *Mg* sequence was aligned by using the MEGA 7.0.26 software and then assigned to a specific *mgc2*-type according to the scheme created by Matucci and collaborators [62]. All the *Mg* isolates with an *mgc2* sequence identical to *Mg* 6/85 were excluded from the study. In order to exclude the presence of ts-11 vaccine strains within the study group, all the *Mg* isolates with an *mgc2* sequence identical to *Mg* ts-11 underwent a further PCR assay [79]. The *Mg* isolates with an amplicon identical in size to the vaccine-specific *gapA* amplicon were excluded from the study. 

### 4.4. MIC Test

The antimicrobial susceptibility of the *Mg* isolates was assessed through MIC testing, carried out by using our internal procedure which is based on Hannan guidelines (with slight modifications) [22], and the standardized method formulated for human mycoplasmas [80]. Briefly, a pure *Mycoplasma* culture of each isolate was obtained after consecutive, in vitro passages, as described by Markey et al. [81] with slight modifications. An additional, final purity check on the *Mycoplasma* culture was performed by using the DGGE technique. The *Mg* suspension was propagated in 6 mL of Avian Mycoplasma medium (Mycoplasma Experience^®^, Reigate, UK) at the third passage on liquid medium. One-hundred µL of this solution at the exponential growth phase of the mycoplasma cells was used for mycoplasma cell titration performed by employing 96-well, u-bottom microtiter plates (Greiner Bio-One, Cassina de Pecchi, Italy). The calculation of the colour changing unit (CCU/mL) was done by using the most probable number method as described in the literature [82]. Once bacterial titre was determined, 1 mL of the initial solution was poured in Avian Mycoplasma liquid medium without inhibitors (Mycoplasma Experience^®^, Reigate, UK) to achieve a standardized inoculum of approximately 10^4^ CCU/mL. The MIC test was performed by employing custom-made 96-well microtiter plates with lyophilized antimicrobials incorporated in (Merlin Diagnostik^®^, lots 140919P95001, 1700630P22001). The list of the antimicrobials present on the MIC plate and their dilution ranges are reported in Table 1. For each work session, a type strain (MG ATCC 15302) with known susceptibility against the tested antimicrobials was tested to ensure MIC data validity and good reproducibility throughout the study. If not identical, type strain MICs were considered to be in essential agreement if they differed of ±1 dilution only. The MIC plates used after the end of 2017 did not contain doxycycline. For each plate, an empty well was filled with sterile liquid medium and considered as a negative control for the test. Another well containing culture broth was filled with the tested *Mg* inoculum and served as positive control. After the *Mg* suspension was poured into the wells, the plates were sealed with a plastic film. The plates were aerobically incubated at 37 ± 1°C and were manually read within 24–48 h, as soon as the positive-control broth indicated mycoplasma growth. Each plate was tested in duplicate. MIC test results were considered valid if both MIC tests generated identical results. For each antimicrobial tested, the MIC value was the lowest antimicrobial concentration able to completely inhibit the growth/metabolism of *Mg* in vitro. In case *Mg* growth/metabolism was not inhibited by the highest/lowest antimicrobial concentration present on the plate, the MIC value was expressed as greater than (>) and as lower than or equal (≤) respectively. MIC50 and MIC90 values, that are the lowest antimicrobial concentration that inhibits the 50% and the 90% of the *Mg* isolates respectively, were calculated. 

### 4.5. Statistical Analyses

The statistical analyses were carried out under R environment [83]. The variation over time of the MIC class frequencies was analyzed by using the asymptotic linear-by-linear association test implemented under a conditional inference framework (package “coin” [84]). This test is similar to the Pearson chi-square test and it is specifically designed to assess linear relationships between ordinal variables, that in our case were the time and the MIC class (MIC value), both expressed as ordered factors. In order to contain type I error inflation, the calculated *p* values were adjusted according to the method of Benjamini and Hochberg (also known as “false discovery rate” method).

The relation between the year of isolation and the single MIC class frequencies of erythromycin, tylosin, tilmicosin, spiramycin, tiamulin, and lincomycin was further characterized by means of two different regression models: a logistic one for erythromycin, because of the depiction of solely two MIC classes ((<0.5, >8 µg/mL) and a proportional odds one (package “ordinal” [85]) for the other antimicrobials. In all cases, the independent variable “year” was a numeric vector obtained from the Z-score transformation of the year of isolation, whereas the dependent variable (the MIC class frequencies) was internally transformed via logit link function. The relationship between the MIC class frequencies of each drug and “year” was assessed by exploring linear, quadratic or cubic forms of the independent variable. The best-fitting model was selected on the basis of the lowest Akaike information criterion value. The goodness of the proportional odds assumptions was verified by the function “scale-test” implemented by the package “ordinal”. Because doxycycline MIC data after 2017 was not available, no statistical analysis for this compound was carried out.

## 5. Conclusions

Collecting MIC data is fundamental for appropriate antimicrobial use and for controlling AMR emergence. The authors expanded the knowledge on antimicrobial sensitivity of *Mg* against different antimicrobial drugs and how this changed over a ten-year time frame. A statistically significant trend toward low MIC-classes was observed for erythromycin, tylosin, tilmicosin, spiramycin, tiamulin, and lincomycin, which means a comeback to susceptibility of the Italian *Mg* isolates toward these drugs. The importance of this data is related also to the fact that recent European data on *Mg* MICs is limited. Future studies are needed to thoroughly comprehend this finding.

## Figures and Tables

**Table 1:** Numerical distribution of the *Mg* isolates within the dilution ranges of the tested antibiotics. The dilution ranges present on the MIC plates are indicated as white boxes in the table. The numbers located in gray boxes indicate those isolates for which growth was not inhibited by the highest concentration present in the MIC plate.

Antibiotic	MIC Values (µg/ML)														
	0.003906	0.0078125	0.015625	0.03125	0.0625	0.125	0.25	0.5	1	2	4	8	16	32	64
Oxytetracycline									9	14	26^50^	9	3^90^	5	1 *
Doxycycline							3	25^50^	18	5^90^					
Enrofloxacin								3	4	5	2	6	9	38^50−90^	
Erythromycin								39^50^					28^90^		
Tilmicosin				31	7^50^		1							4	24^90^
Tylosin		6	14	15^50^	3	1		3	2	5	11^90^	1	1	1	4
Spiramycin								39^50^				1	2	25^90^	
Tiamulin		25	13^50^	6	11	8^90^	4								
Lincomycin									1	6	20	6	12^50^	11	11^90^
Florfenicol								5	27	32^50−90^	3				

The MIC values are expressed in μg/mL. The dilution range of each antibiotic is represented by the grey cells (e.g., enrofloxacin was tested from 0.125 to 16 μg/mL). Superscript numbers indicate the MIC50 and MIC90 values. * Isolate numbers outside dilution range were not inhibited by the highest concentration of antibiotic.

**Table 2:** Results of the asymptotic linear-by-linear association test of MIC-class frequency vs. year.

Antibiotic	*Z*-value	*p*-Value	Adjusted *p*-Value
Oxytetracycline	−0.97	0.33	0.38
Enrofloxacin	−1.53	8.65⋅10^−2^	0.11
Erythromycin	−3.14	1.71⋅10^−3^	5.12⋅10^−3^
Tilmicosin	−3.95	7.87⋅10^−5^	3.54⋅10^−4^
Tylosin	−2.82	4.84⋅10^−3^	1.09⋅10^−2^
Spiramycin	−2.65	7.97⋅10^−3^	1.43⋅10^−2^
Tiamulin	−4.18	2.94⋅10^−5^	2.65⋅10^−4^
Lyncomycin	−2.57	1.03⋅10^−2^	1.55⋅10^−2^
Florfenicol	0.45	0.65	0.65

## Supplementary Materials

The following are available online at www.mdpi.com/article/10.3390/antibiotics11081021/s1, Figure S1: Graphical distribution of the *Mg* isolates along the dilution range (expressed in μg/mL) of the ten antimicrobials included in the study. The different concentrations of antimicrobial used in the study are reported on the horizontal axis of abscissas while the number of the isolates that were inhibited by each antimicrobial concentration is reported on the vertical axis of the ordinates. The concentration that inhibits the 50% of the isolates (MIC50) is indicated as a grey bar; the concentration that inhibits the 90% of the isolates (MIC90) is indicated as a black bar. Table S1: Parameter estimates with relative standard error (Std.Error), Wald statistic (*Z*-value), and *p*-value of the logistic model relating the frequency of observation of the highest erythromycin MIC classes (>8 µg/mL) to the cubic of the variable year. Table S2: Parameter estimates with relative standard error (Std.Error), Wald statistic (*Z*-value), and *p*-value of the proportional odds model relating the frequency of observation of the different lyncomycin MIC value classes to the cubic of the variable year. Table S3: Parameter estimates with relative standard error (Std.Error), Wald statistic (*Z*-value), and *p*-value of the proportional odds model relating the frequency of observation of the different spiramycin MIC value classes to the variable year. Table S4: Parameter estimates with relative standard error (Std.Error), Wald statistic (*Z*-value), and *p*-value of the proportional odds model relating the frequency of observation of the different tiamulin MIC value classes to the variable year. Table S5: Parameter estimates with relative standard error (Std.Error), Wald statistic (*Z*-value), and *p*-value of the proportional odds model relating the frequency of observation of the different tylosin MIC value classes to the cubic of the variable year. Table S6: Parameter estimates with relative standard error (Std.Error), Wald statistic (*Z*-value), and *p*-value of the proportional odds model relating the frequency of observation of the different tilmicosin MIC value classes to the cubic of the variable year. Table S7: Mg isolates sorted per year of isolation and animal species.
