# Supplementary material for: The Monitoring of Mycoplasma gallisepticum Minimum Inhibitory Concentrations during the Last Decade (2010–2020) Seems to Reveal a Comeback of Susceptibility to Macrolides, Tiamulin, and Lincomycin
Source: Antibiotics (Basel). 2022 Jul 29;11(8):1021. doi: 10.3390/antibiotics11081021 (PMC9404793; doi:10.3390/antibiotics11081021)
Supplement: Supplementary file 1 [file antibiotics-11-01021-s001.zip › antibiotics-1820163-supplementary.pdf]

Table S1. Parameter estimates with relative standard error (Std.Error), Wald statistic (Z-value) and p-value of the logistic model relating the frequency of observation of the highest erythromycin MIC classe (>8 µg/ml) to the cubic of the variable year.

<table>
<tr><th></th><th>Estimate</th><th>Std. Error</th><th>Z-value</th><th>p-value</th></tr>
<tr><td>Coefficients</td><td></td><td></td><td></td><td></td></tr>
<tr><td>Intercept</td><td>-0.317</td><td>0.272</td><td>-1.164</td><td></td></tr>
<tr><td>year^3</td><td>-0.568</td><td>0.221</td><td>-2.570</td><td>$1.02\cdot10^{-2}$</td></tr>
</table>

Table S2. Parameter estimates with relative standard error (Std.Error), Wald statistic (Z-value) and p-value of the proportional odds model relating the frequency of observation of the different lyncomycin MIC value classes to the cubic of the variable year.

<table>
<tr><th></th><th>Estimate</th><th>Std. Error</th><th>Z-value</th><th>p-value</th></tr>
<tr><td>Coefficients</td><td></td><td></td><td></td><td></td></tr>
<tr><td>year^3</td><td>-0.294</td><td>0.089</td><td>-3.305</td><td>$9.50\cdot10^{-4}$</td></tr>
<tr><td>Threshold coefficients</td><td></td><td></td><td></td><td></td></tr>
<tr><td>1|2</td><td>-4.713</td><td>1.053</td><td>-4.474</td><td></td></tr>
<tr><td>2|4</td><td>-2.505</td><td>0.449</td><td>-5.582</td><td></td></tr>
<tr><td>4|8</td><td>-0.525</td><td>0.263</td><td>-1.994</td><td></td></tr>
<tr><td>8|16</td><td>-0.124</td><td>0.256</td><td>-0.483</td><td></td></tr>
<tr><td>16|32</td><td>0.698</td><td>0.269</td><td>2.594</td><td></td></tr>
<tr><td>32|>32</td><td>1.678</td><td>0.338</td><td>4.961</td><td></td></tr>
</table>

Table S3. Parameter estimates with relative standard error (Std.Error), Wald statistic (Z-value) and p-value of the proportional odds model relating the frequency of observation of the different spiramycin MIC value classes to the variable year.

<table>
<tr><th></th><th>Estimate</th><th>Std. Error</th><th>Z-value</th><th>p-value</th></tr>
<tr><td>Coefficients</td><td></td><td></td><td></td><td></td></tr>
<tr><td>year</td><td>-0.647</td><td>0.274</td><td>-2.363</td><td>$1.81\cdot10^{-2}$</td></tr>
<tr><td>Threshold coefficients</td><td></td><td></td><td></td><td></td></tr>
<tr><td><0.5|8</td><td>0.345</td><td>0.264</td><td>1.306</td><td></td></tr>
<tr><td>8|16</td><td>0.416</td><td>0.266</td><td>1.563</td><td></td></tr>
<tr><td>16|>16</td><td>0.561</td><td>0.270</td><td>2.076</td><td></td></tr>
</table>

Table S4. Parameter estimates with relative standard error (Std.Error), Wald statistic (Z-value) and p-value of the proportional odds model relating the frequency of observation of the different tiamulin MIC value classes to the variable year.

<table>
<tr><th></th><th>Estimate</th><th>Std. Error</th><th>Z-value</th><th>p-value</th></tr>
<tr><td>Coefficients</td><td></td><td></td><td></td><td></td></tr>
<tr><td>year</td><td>-1.154</td><td>0.268</td><td>-4.309</td><td>$1.64\cdot10^{-5}$</td></tr>
<tr><td>Threshold coefficients</td><td></td><td></td><td></td><td></td></tr>
<tr><td><0.0078125|0.0156</td><td>-0.818</td><td>0.296</td><td>-2.765</td><td></td></tr>
<tr><td>0.0156|0.03125</td><td>0.214</td><td>0.275</td><td>0.778</td><td></td></tr>
<tr><td>0.03125|0.0625</td><td>0.698</td><td>0.286</td><td>2.443</td><td></td></tr>
<tr><td>0.0625|0.125</td><td>1.776</td><td>0.353</td><td>5.037</td><td></td></tr>
<tr><td>0.125|0.25</td><td>3.145</td><td>0.545</td><td>5.771</td><td></td></tr>
</table>

Table S5. Parameter estimates with relative standard error (Std.Error), Wald statistic (Z-value) and p-value of the proportional odds model relating the frequency of observation of the different tylosin MIC value classes to the cubic of the variable year.

	Estimate	Std. Error	Z-value	p-value
**Coefficients**				
$year^3$	-0.326	0.121	-2.686	$7.24 \cdot 10^{-3}$
**Threshold coefficients**				
<0.0625|0.0625	0.131	0.258	0.507	
0.0625|0.125	0.271	0.260	1.043	
0.125|0.5	0.343	0.262	1.309	
0.5|1	0.560	0.268	2.087	
1|2	0.705	0.274	2.575	
2|4	1.078	0.292	3.696	
4|8	2.288	0.417	5.491	
8|16	2.467	0.445	5.543	
16|32	2.667	0.481	5.549	
32|>32	2.906	0.529	5.488	

Table S6. Parameter estimates with relative standard error (Std.Error), Wald statistic (Z-value) and p-value of the proportional odds model relating the frequency of observation of the different tilmicosin MIC value classes to the cubic of the variable year.

	Estimate	Std. Error	Z-value	p-value
**Coefficients**				
$year^3$	-0.804	0.223	-3.607	$3.10 \cdot 10^{-4}$
**Threshold coefficients**				
<0.03125|0.03125	-1.342	0.335	-4.004	
0.03125|0.0625	-0.148	0.274	-0.543	
0.0625|0.25	0.357	0.275	1.300	
0.25|32	0.431	0.277	1.557	
32|>32	0.732	0.286	2.555	

Table S7. *Mg* isolates sorted per year of isolation and animal species.

Year of isolation	Chicken	Turkey	Other	Total
2010	-	3	-	3
2011	1	3	-	4
2012	2	3	2	7
2013	8	4	2	14
2014	3	7	-	10
2015	2	1	1	4
2016	7	2	-	9
2017	1	2	-	3
2018	4	3	1	8
2019	2	-	-	2
2020	2	1	-	3
Total	32	29	6	67

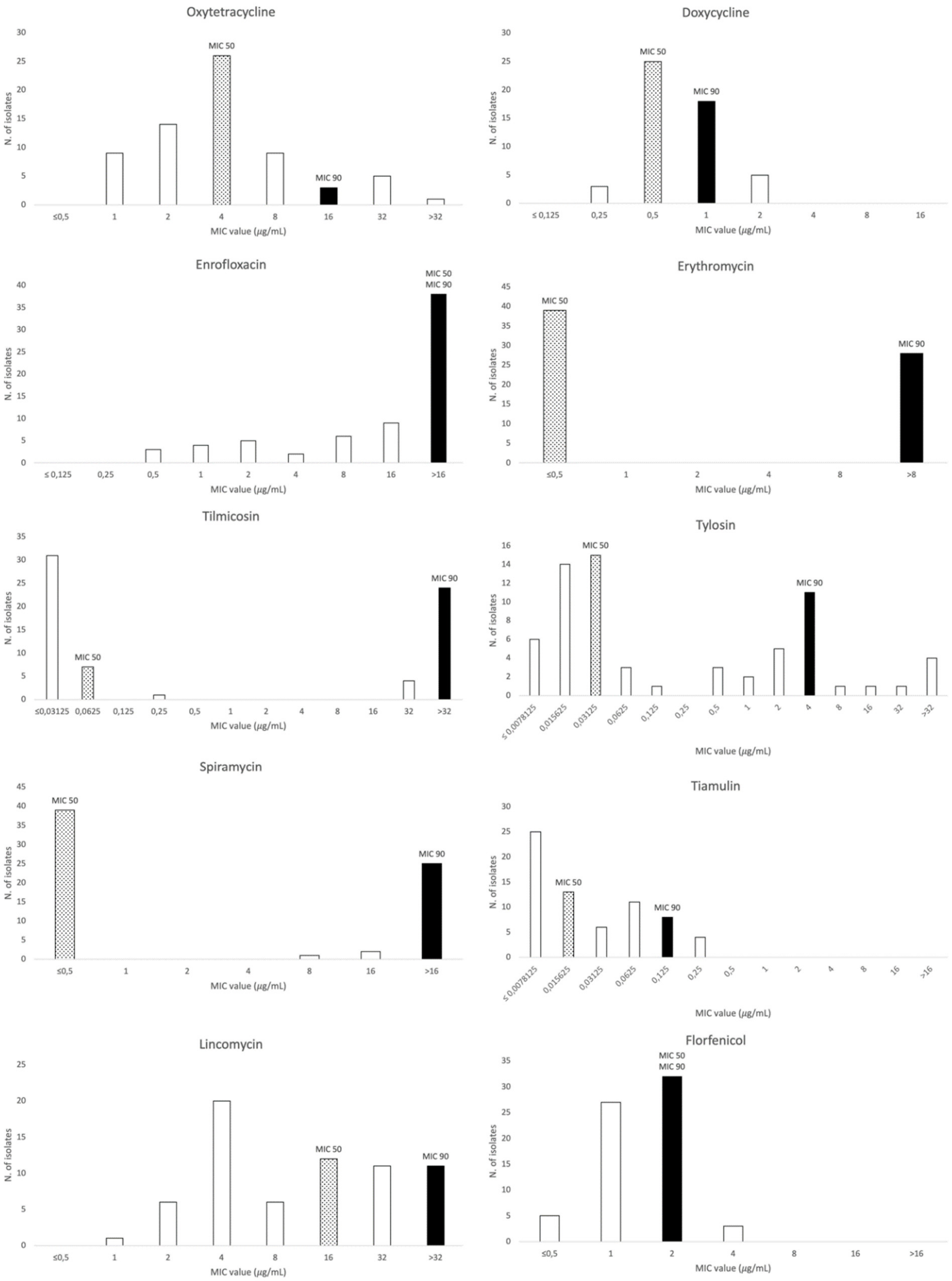

Figure S1. Graphical distribution of the *Mg* isolates along the dilution range (expressed in μg/mL) of the ten antimicrobials included in the study. The different concentrations of antimicrobial used in the study are reported on the horizontal axis of abscissas while the number of the isolates that were inhibited by each antimicrobial concentration is reported on the vertical axis of the ordinates. The concentration that inhibits the 50% of the isolates (MIC50) is indicated as a grey bar; the concentration that inhibits the 90% of the isolates (MIC90) is indicated as a black bar.
